# Supplementary material for: Determinants of mammography screening participation–a cross-sectional analysis of the German population-based Gutenberg Health Study (GHS)
Source: PLoS One. 2022 Oct 5;17(10):e0275525. doi: 10.1371/journal.pone.0275525 (PMC9534433; doi:10.1371/journal.pone.0275525)
Supplement: Attachment — Submitted filename: Response to reviewers.docx [file pone.0275525.s001.docx]

Thank you for reviewing our manuscript. **Please find attached our point-by-**point response **to the comments**. Reviewer comments **are** in black font, Author replies in black font italic and underlined.

1. Is the manuscript technically sound, and do the data support the conclusions?

The manuscript must describe a technically sound piece of scientific research with data that supports the conclusions. Experiments must have been conducted rigorously, with appropriate controls, replication, and sample sizes. The conclusions must be drawn appropriately based on the data presented.

Reviewer #1: Yes

Reviewer #2: Yes

2. Has the statistical analysis been performed appropriately and rigorously?

Reviewer #1: I Don't Know

Reviewer #2: I Don't Know

3. Have the authors made all data underlying the findings in their manuscript fully available?

The [PLOS Data policy](http://www.plosone.org/static/policies.action#sharing) requires authors to make all data underlying the findings described in their manuscript fully available without restriction, with rare exception (please refer to the Data Availability Statement in the manuscript PDF file). The data should be provided as part of the manuscript or its supporting information, or deposited to a public repository. For example, in addition to summary statistics, the data points behind means, medians and variance measures should be available. If there are restrictions on publicly sharing data—e.g. participant privacy or use of data from a third party—those must be specified.

Reviewer #1: Yes

Reviewer #2: No

4. Is the manuscript presented in an intelligible fashion and written in standard English?

PLOS ONE does not copyedit accepted manuscripts, so the language in submitted articles must be clear, correct, and unambiguous. Any typographical or grammatical errors should be corrected at revision, so please note any specific errors here.

Reviewer #1: Yes

Reviewer #2: Yes

5. Review Comments to the Author

Please use the space provided to explain your answers to the questions above. You may also include additional comments for the author, including concerns about dual publication, research ethics, or publication ethics. (Please upload your review as an attachment if it exceeds 20,000 characters)

Reviewer #1: It is a technically sound study, well conducted and a well written manuscript. Would be nice to add a little detail about the mammography invitation process for the non-German reader.

*Answer: We added in the introduction (line 67ff) a part about the invitation process.*

*“Eligible women are informed every two years by an invitation letter from one of 14 Central Offices which organize the mammography screening program nationwide. A detailed information brochure is sent out with the invitation. The brochure serves as a decision-making aid and provides information on the examination procedure, on breast cancer, and on possible advantages as well as disadvantages of participating in a screening program.”*

I feel it would be important to explore perhaps as a separate study, the reasons behind the decrease in mammography (recently) with higher education. Are they accessing bad information on social media or misinterpreting information about radiation exposure etc. This may inform further decisions and the invitation process may need to have more detailed information on dispelling the myths.

*Answer: We agree with the reviewer that specifically the groups that do not participate would need to be examined more closely in a qualitative study. It would also be interesting to find out where these groups get their information.*

Reviewer #2: The article addresses an important matter regarding participation in screening mammography programs. Participation in screening programs is mostly affected by knowledge/awareness, access and personal beliefs. Being a smoker or alcohol consumer, BMI etc can have a direct relation with increasing breast cancer risk, but PARTICIPATING or not participating in a free mammography screening program due to these factors doesn't make sense.

*Answer: In the absence of better variables, we included the variables smoking, BMI, alcohol consumption, etc. as a proxy for health behaviors in our model.*

Also p-values and data coul*d not be found so exact significance of these variables could not be ascertained.*

*Answer: We understand the concerns from the reviewer. We do not report p-values anywhere in the manuscript. The reasons for doing so:*

1. *The sample size is sufficiently large that even seemingly trivial differences are likely to be statistically significant.*
2. *P-values are commonly misinterpreted as the probability that the test hypothesis is true, or as the probability that observed association is due to chance alone. Both are false.*

*In sum, in the present study we do not believe that adding P values would help readers to get a better sense of whether the reported characteristics differ across women, or that p-values and significance testing would add valuable information beyond the reported confidence intervals.*

*We hope that the explanations will satisfy the reviewers and that the editors will appreciate our efforts to conform with the STOBE statement and the position of the American Statistical Association (2016). Enclosed we send the result tables with the p-values.*

*Models 1-3*

*Model 4*

*Model 5*

*Thank you for reviewing our manuscript and for the helpful comments. We are looking forward to your reply.*

*best regards on behalf of all authors*

*Roman Pokora*
